# CHILD-IVITAB, a Novel Orodispersible Ivermectin Formulation—Pharmaceutical Evaluation and Clinical Acceptability in Children <15 kg

**DOI:** 10.3390/pharmaceutics18091147

**Published:** 2026-09-11

**Authors:** Diell Aliu, Thibault Vallet, Jörg Huwyler, Maxim Puchkov, Lorenna Pereira de Souza, Eline Freitas Medeiros, Wuelton Monteiro, Jacqueline de Almeida Gonçalves Sachett, Fabrice Ruiz, Michael Buettcher, Marc Pfister, Lorenz von Seidlein, Joel Tarning, Kevin C. Kobylinski

**Affiliations:** 1 Department of Pharmaceutical Sciences, University of Basel, 4001 Basel, Switzerland; diell.aliu@unibas.ch (D.A.); joerg.huwyler@unibas.ch (J.H.); maxim.puchkov@unibas.ch (M.P.); 2 ClinSearch, 92240 Malakoff, France; thibault.vallet@clinsearch.net (T.V.); fabrice.ruiz@clinsearch.net (F.R.); 3 Fundação de Medicina Tropical Doutor Heitor Vieira Dourado, Manaus 69040-000, Brazil; lorennapsouza@gmail.com (L.P.d.S.); elinenaiane@gmail.com (E.F.M.); wmonteiro@uea.edu.br (W.M.); jsachett@uea.edu.br (J.d.A.G.S.); 4 Escola Superior de Ciências da Saúde, Universidade do Estado do Amazonas, Manaus 69065-001, Brazil; 5 Fundação Hospitalar Alfredo da Matta, Manaus 69065-130, Brazil; 6 University Children’s Hospital Basel (UKBB), 4031 Basel, Switzerland; michael.buettcher@ukbb.ch (M.B.); marc.pfister@ukbb.ch (M.P.); 7 Faculty of Health Sciences and Medicine, University of Lucerne, 6002 Lucerne, Switzerland; 8 Paediatric Infectious Diseases Unit, Children’s Hospital of Central Switzerland (Kidz), 6004 Lucerne, Switzerland; 9 Mahidol Oxford Tropical Medicine Research Unit, Bangkok 10400, Thailand; lorenz@tropmedres.ac (L.v.S.); joel@tropmedres.ac (J.T.); 10 Nuffield Department of Medicine, University of Oxford, Oxford OX3 7LF, UK

**Keywords:** ivermectin, scabies, CHILD-IVITAB, orodispersible tablet, pediatric drug delivery, Template Inverted Particle, ClinSearch Acceptability Score Test

## Abstract

**Background/Objectives**: Scabies is a disease that disproportionately affects young children. Oral ivermectin is a mainstay of scabies therapy, yet children <15 kg are excluded due to insufficient safety data and the lack of age-appropriate formulations. To address this gap, we developed CHILD-IVITAB, a 1 mg ivermectin orodispersible tablet based on the Template Inverted Particle technology, which enables rapid disintegration and intrinsic taste masking in a mechanically robust tablet. **Methods**: We report the pharmaceutical characterization of CHILD-IVITAB tablets. The EPIC-15 trial (NCT06404333) was a randomized clinical trial administering CHILD-IVITAB to children weighing 5 to <15 kg with uncomplicated scabies in Brazil. Acceptability was assessed at two administrations (D0, D7) using the ClinSearch Acceptability Score Test (CAST^®^), wherein CHILD-IVITAB was compared to a reference dataset. **Results**: The 40 mg tablets had a tensile strength of 1.87 ± 0.16 MPa, and met Ph. Eur. requirements for uniformity of mass and dosage units. CHILD-IVITAB tablets disintegrated in vitro in 18.3 ± 1.1 s and released >50% of the dose within 1 min in artificial saliva. Thirteen children were enrolled in the trial. The full dose was taken in 100% of the 26 evaluations, 73% of administrations elicited a neutral or positive reaction, and no dosage form alteration, food/drink co-administration, or extra device was required. The barycenter of all evaluations fell within the positive zone of the CAST^®^ framework. **Conclusions**: These results support CHILD-IVITAB as a child-friendly ivermectin formulation suitable for children 5 to <15 kg, a population currently excluded from ivermectin treatment and prevention programs.

## 1. Introduction

Scabies is a skin infestation caused by the mite *Sarcoptes scabiei*. It is characterized by severe itching and is transmitted through prolonged direct skin-to-skin contact. Scabies is a major public health problem, especially in resource-poor populations, and was recognized as a neglected tropical disease (NTD) by the WHO in 2017 [1]. A systematic analysis of the Global Burden of Disease Study 2021 estimated a global incidence of 622.5 million cases and 5.3 million disability-adjusted-life years (DALYs), disproportionately affecting young children, with the highest incidence rate observed in children <5 years of age [2].

Ivermectin is a broad-spectrum antiparasitic drug known to be safe and effective in the treatment of scabies [3,4]. More than 400 million treatments are distributed annually by mass drug administration (MDA), with more than 6 billion treatments administered to date [5]. Despite the well-established safety profile of ivermectin in persons weighing more than 15 kg, ivermectin is currently not indicated for use in children weighing less than 15 kg due to insufficient safety data in this population [6].

Currently, the only commercially available oral ivermectin formulations are tablets designed for persons >15 kg that can readily swallow tablets. In children <15 kg, it is recommended that ivermectin tablets be crushed and suspended in liquid or mixed with food prior to administration [7], or given as a syrup [8]. The procedure of crushing and suspension is prone to imprecise dosing, loss of product during suspension, handling difficulties, and is not practical at scale for MDA. These limitations highlight the need for a child-friendly ivermectin formulation that ensures accurate dosing, intrinsic taste masking, acceptable palatability, stability in hot and humid environments, and suitability for use in both clinical and MDA settings. To address this need, a novel orodispersible tablet (ODT) formulation of ivermectin, called CHILD-IVITAB, has been developed. It is based on the Template Inverted Particle (TIP) technology, which consists of calcium phosphate microcapsules that serve as multifunctional drug carriers [9]. These TIP tablets offer a unique combination of hard tablets with rapid disintegration and enhanced taste masking. Their high internal porosity enables efficient loading of a wide range of drug substances, while the porous shell facilitates fast water uptake upon contact with saliva, resulting in in vitro disintegration times of less than 20 s [10]. This makes CHILD-IVITAB particularly suitable for pediatric patients, who commonly have difficulties swallowing conventional tablets. Dao et al. evaluated CHILD-IVITAB in a phase I crossover trial in 16 healthy adults and found enhanced palatability, good tolerability, and a more controlled absorption profile compared to standard ivermectin tablets (Stromectol^®^, MSD) [11]. Golhen et al. developed a population pharmacokinetic model, which indicated that a dose of 250 μg/kg CHILD-IVITAB in children <15 kg should achieve equivalent ivermectin exposure to the standard 200 μg/kg dose of STROMECTOL^®^ in adults and children ≥15 kg [12].

In this paper, we report the pharmaceutical characterization and clinical acceptability of the 1 mg CHILD-IVITAB ODT that was administered to children weighing 5 to less than 15 kg with uncomplicated scabies in a phase IIb study conducted in Brazil. Acceptability, a prespecified secondary outcome, was formally evaluated in accordance with European Medicines Agency (EMA) recommendations for pediatric medicines [13,14], using the standardized ClinSearch Acceptability Score Test (CAST^®^) [15]. Tablet performance was assessed in terms of tensile strength, in vitro disintegration time, dissolution performance, uniformity of mass, and uniformity of dosage units.

## 2. Materials and Methods

### 2.1. CHILD-IVITAB Tablet Manufacture

Ivermectin API was purchased from Pharmaserv (Stansstad, Switzerland). Citric acid monohydrate and ethanol were purchased from Hänseler (Herisau, Switzerland). Sodium croscarmellose was obtained from Spectrum (New Brunswick, NJ, USA). Rebaudioside A and peach aroma powder were purchased from Fontana (Canosa di Puglia, Italy). The microcapsule material (TIP) was manufactured at the Hospital Pharmacy in Basel according to the method described by Kost et al. [9].

The production of the study material took place under GMP at the Hospital Pharmacy in Basel, Switzerland. Ivermectin was dissolved in ethanol; the resulting solution was mixed with TIP powder and dried in an open container in a fume hood at room temperature for 24 h. Residual ethanol and moisture were controlled by loss on drying (<0.5%). This resulted in ivermectin-loaded TIP at a drug load of 2.8% (*w*/*w*), which was combined with citric acid monohydrate (5% *w*/*w*), croscarmellose sodium (3% *w*/*w*), rebaudioside A (2.5% *w*/*w*), and peach aroma powder (0.5% *w*/*w*) to obtain a final tablet with a target dose of 1 mg ivermectin. Taste masking was achieved by both incorporation of ivermectin within TIP microcapsules as well as addition of aroma.

The tablets were compacted using a STYL’One Evo (Medelpharm, Beynost, France) with 5 mm flat-faced tooling. The tablets had a target mass of 40 mg, containing 1 mg of ivermectin. In total, a batch of 950 tablets was produced and the uniformity of mass of all tablets was measured with a SADE SP-B60 checkweigher (CI Precision, Salisbury, UK). Tablets were packaged in 30 mL wide-mouth amber glass jars, 20 tablets per jar, closed with white tamper-evident screw caps. Tablets were stored at 15–30 °C.

### 2.2. Tablet Hardness and Tensile Strength

Tablet hardness (n = 6) and diameter were measured using a hardness tester (Pharmatron, Thun, Switzerland). Tablet height was measured with a CS-15CPX caliper (Mitutoyo, Kawasaki, Japan). Tensile strength was calculated according to Equation (1) described by Fell and Newton [16]:

(1) $$\sigma T=\frac{2F}{\pi dh}$$

where *σ_T_* is the tensile strength (MPa), *F* is the crushing force (N), *d* is the diameter (mm) of the tablets, and *h* is the height (mm) of the tablet. In line with EMA recommendations on age-appropriate formulation design [13] and acceptability data for solid oral dosage forms in young children [17], tablets were designed to be small in all dimensions, with a diameter of ≤5 mm and a thickness of ≤1.5 mm. A minimum tensile strength of 1.7 MPa is required to ensure stability during transport and storage [18].

### 2.3. In Vitro Disintegration and Dissolution Testing

In vitro disintegration (n = 6) was assessed using a Sotax DT2 (Sotax, Aesch, Switzerland), which complies with the pharmacopeial method described in Ph. Eur. 2.9.1. The experiment was carried out at 37 °C, with 850 mL of water, and a frequency of 30 cycles per minute. Dissolution of single tablets (n = 6) was measured in 900 mL of artificial saliva (AFS) fluid at pH 6.8, following previously published methodology [10]. AFS dissolution medium was prepared by combining 1017 mg sodium chloride, 61 mg magnesium chloride hexahydrate, 204 mg sodium phosphate dibasic heptahydrate (Carl Roth GmbH, Karlsruhe, Germany), 228 mg calcium chloride dihydrate, 676 mg potassium carbonate sequihydrate, and 273 mg sodium phosphate monobasic monohydrate (Merck, Darmstadt, Germany) per liter of aqueous solution. The pH was adjusted to 6.8 by titrating with 0.1 N hydrochloric acid (HCl). To ensure sink conditions, sodium lauryl sulfate (SLS, 0.5% *w*/*v*) (Carl Roth GmbH, Karlsruhe, Germany) was added to the AFS dissolution medium, as recommended in the USP ivermectin monograph [19].

The experiment was performed with a Sotax AT7 (Sotax, Aesch, Switzerland) according to the paddle apparatus method described in Ph. Eur. 2.9.3 with a paddle speed of 100 rpm. Samples of 1 mL were drawn at 1, 2, 3, 5, 10, 20, 30, and 60 min and replaced with 1 mL of fresh dissolution medium. A final sample was collected at 65 min after increasing the paddle speed to 200 rpm for 5 min, to ensure complete drug release.

### 2.4. Ivermectin Quantification

Ivermectin was quantified on a Shimadzu Nexera X2 UPLC system (Shimadzu, Kyoto, Japan) with the UV detector set to 245 nm. Phenomenex Luna C18 column (3 μm, 50 × 4.6 mm) at 50 °C was used for chromatographic separation. The mobile phase consisted of acetonitrile, methanol, and water (53:35:12 *v*/*v*/*v*) with a flow rate of 1.2 mL/min. Samples were centrifuged at 8000 rcf for 5 min prior to injection. The injection volume was 20 μL, with a total run time of 10 min.

Uniformity of dosage units was assessed by content uniformity testing in accordance with Ph. Eur. 2.9.40. The ivermectin from tablets (n = 10) was extracted in methanol; samples were diluted to 0.05 mg/mL and filtered through 0.45 um PTFE filters prior to injection.

Ivermectin was quantified using a LaChrom Elite HPLC system (Hitachi, Tokyo, Japan) equipped with DAD detector set to 245 nm, Waters Symmetry reversed-phase C18 column (3.5 μm, 100 × 4.6 mm) at 30 °C. The mobile phase consisted of acetonitrile, methanol, and water (53:35:12, *v*/*v*/*v*) with an isocratic flow of 1.2 mL/min. The injection volume was 20 μL and the total run time was 12 min.

### 2.5. Study Participants and Trial Design

The Evaluating Pediatric Ivermectin in Children Under 15 kg (EPIC-15) trial was a prospective, randomized, open-label, phase IIB trial conducted in Manaus, Brazil, at the Fundação Hospitalar Alfredo da Matta. Main inclusion criteria included children aged at least 3 months, weighing 5 to less than 15 kg, with uncomplicated scabies infestation, and in otherwise good health. Uncomplicated scabies was diagnosed according to the International Alliance to Control Scabies consensus criteria for the diagnosis of scabies [20]. Main exclusion criteria included personal history of renal or hepatic impairment; severe malnutrition; crusted scabies or severe bacterial infection (e.g., sepsis); taken ivermectin within last two weeks; known allergy to ivermectin; use of prescription or non-prescription drugs, vitamins, herbs, or supplements especially CYP3A4 inhibitors and inducers; history of travel to West or Central Africa; or being born prematurely. A full list of the inclusion and exclusion criteria along with study description can be found at ClinicalTrials.gov (NCT06404333). The sample size was planned to be 66 participants, based on the primary study objective on pharmacokinetics. The primary outcome was pharmacokinetics, while secondary outcomes included safety, efficacy, and acceptability. Pharmacokinetic, safety, and efficacy outcomes will be reported elsewhere.

Children were randomly assigned to one of two ivermectin regimens: 200 μg/kg or 400 μg/kg. Randomization to regimen was pre-assigned by a trial statistician. Randomization allocation were concealed in envelopes and assigned sequentially to subject. Only the trial statistician and pharmacist had access to the random allocation sequence. Treatments were administered twice, on days 0 (D0) and 7 (D7). The number of tablets (1 mg) per administration was determined by child weight and dose arm, ranging from 1 to 6 tablets per subject. The child was provided a small amount of water to ingest prior to tablet administration. The tablets were placed into the buccal pouch of the child, where they disintegrated into a white paste. The endpoint for disintegration is reached when only a white paste remains. The presence of the white paste formation was directly observed by the study nurses. When more than one tablet was administered, the dose was divided between the left and right buccal pouch, in alternating application. If three or more tablets were administered, then the previous tablet on that side of the mouth had to disintegrate prior to administering the next tablet in the same location. The dose was defined as fully taken when the final tablet disintegrated, reflecting observed completion of administration rather than confirmed quantitative ingestion. The time from start to the final tablet disintegrating was recorded.

### 2.6. Acceptability Scoring

Acceptability was evaluated using the ClinSearch Acceptability Score Test (CAST^®^) [15] at both treatment administrations (D0 and D7). For each administration, a trained study team member recorded nine observational variables: results of intake (fully, partly, or not taken), patient reaction during administration (positive, neutral, or negative on a three-point facial hedonic scale), preparation and administration time (rounded to the nearest 10-second interval, with a minimum of 10 s), and whether any of six possible methods were used to improve administration (dividing the dose, altering the dosage form, co-administering food/drink, using an extra device, promising a reward, or using physical restraint such as the parent holding the child in a specific position for administration). Combined preparation and administration time were categorized as short (≤1 min), medium (>1 to ≤2.5 min), or long (>2.5 min).

**Figure 1:**
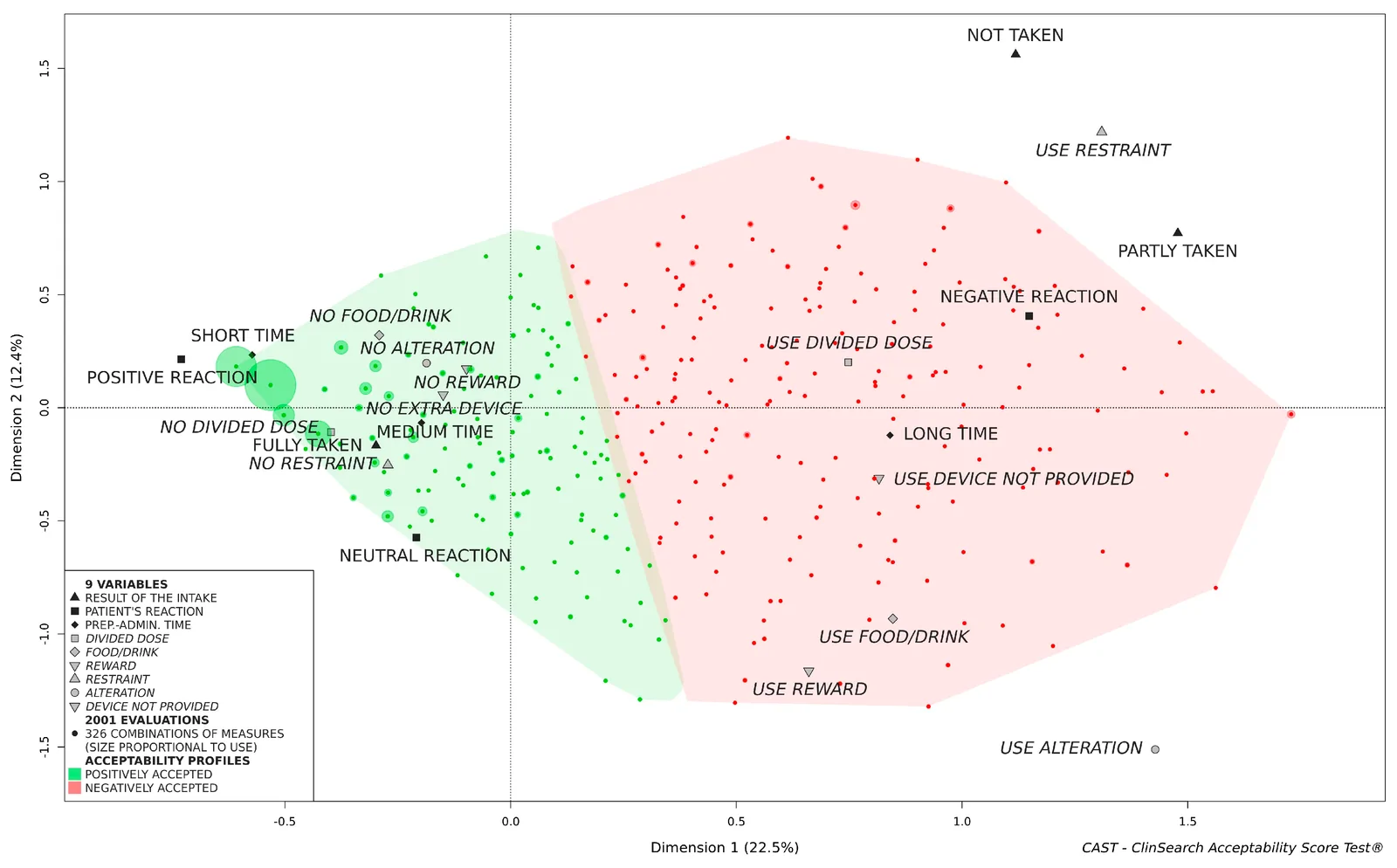
CAST acceptability reference framework defining the positive (green) and negative (red) zones for pediatric oral medicines.

These evaluations were mapped onto an acceptability reference framework derived from a standardized dataset of 2001 evaluations of various oral medicines in children under 12 years of age across nine countries. This dataset contains no repeated measures, with a single evaluation for each patient–drug combination. Using Multiple Correspondence Analysis and hierarchical clustering, this framework defines two areas: a positive zone (green) and a negative zone (red) (Figure 1).

CHILD-IVITAB was positioned on the framework at the barycenter of all collected evaluations, with 90% confidence ellipses. The confidence ellipses were generated by bootstrap resampling and encompass 90% of the bootstrap-estimated barycentres, providing a distribution-free graphical representation of result stability and uncertainty. A medicine can be classified as positively accepted if both the barycenter and the entire confidence ellipse fall within the positive zone.

The acceptability of CHILD-IVITAB was compared to the average acceptability score of conventional tablets in children of the same age range from the dataset that led to the acceptability reference framework. This subset comprised 80 evaluations covering 44 medicinal products corresponding to 33 drugs from 21 therapeutics, collected in India and Morocco. This comparison served as a surrogate for the acceptability of currently marketed oral dosage forms of ivermectin, for which no standardized acceptability data exist in this age group.

Statistical comparisons of observational variables between dose groups and subpopulations were performed using Pearson’s chi-squared test, Fisher’s exact test, exact McNemar test, or Wilcoxon signed-rank test, as appropriate. Subgroup analyses were then similarly performed. The evaluations were subsequently partitioned into subgroups depending on the factors of interest to investigate their influence on acceptability: timepoints (D0 vs. D7), the treatment regimen (200 vs. 400 µg/kg), as well as the sex (female vs. male) and the age group (<1 year vs. ≥1 year) of subjects.

A minimum of 30 evaluations was required to obtain a reliable acceptability score using the CAST^®^ methodology. Below this threshold, we only describe acceptability tendency. Furthermore, we positioned onto the reference framework only subsets with more than 10 evaluations. Below this arbitrary threshold, interest is very limited as reliability of the score is low and confidence ellipses are often large.

Acceptability analyses were performed using R (RStudio 2025.09.0 Build 387). The R package FactoMineR [21] was used to perform multivariate analysis.

## 3. Results

### 3.1. Tablet Characterization and Performance

The CHILD-IVITAB ODT was characterized in terms of its mechanical properties, disintegration behavior, dissolution performance, and uniformity of mass. Tensile strength and in vitro disintegration time were measured with six tablets from the clinical batch. The results are summarized in Table 1. The tensile strength was 1.87 MPa (RSD 8.6%), indicating sufficient mechanical robustness for handling, packaging, and transport. The in vitro disintegration time was 18.3 s (RSD 6.1%), well within the pharmacopeial limit of 180 s and the 30 s limit described by the United States Food and Drug Administration [22].

The mass of all 950 tablets in the clinical batch was individually determined (Table 1). The mean tablet mass was 40.55 mg (RSD 1.1%), ranging from 38 to 42 mg. All individual tablet masses were within ±10% acceptance criterion of the Ph. Eur. 2.9.5 for tablets of average mass ≤ 80 mg. Content uniformity was determined with the LaChrom HPLC method (Section 2.1) using 10 tablets from the clinical batch (Table 1). The mean ivermectin content was 1.00 ± 0.02 mg, representing 100% of the label claim (range of 0.98–1.05 mg). All individual values fell within the pharmacopeial limits of 85–115%, and the acceptance value (AV) of 5.5 was well below the maximum value of 15.0. The batch complied with the requirements of Ph. Eur. 2.9.40 [23]. The tablets met all pharmacopeial and design criteria defined in Section 2, including the dimensional and mechanical target to support pediatric patient compliance.

**Figure 2:**
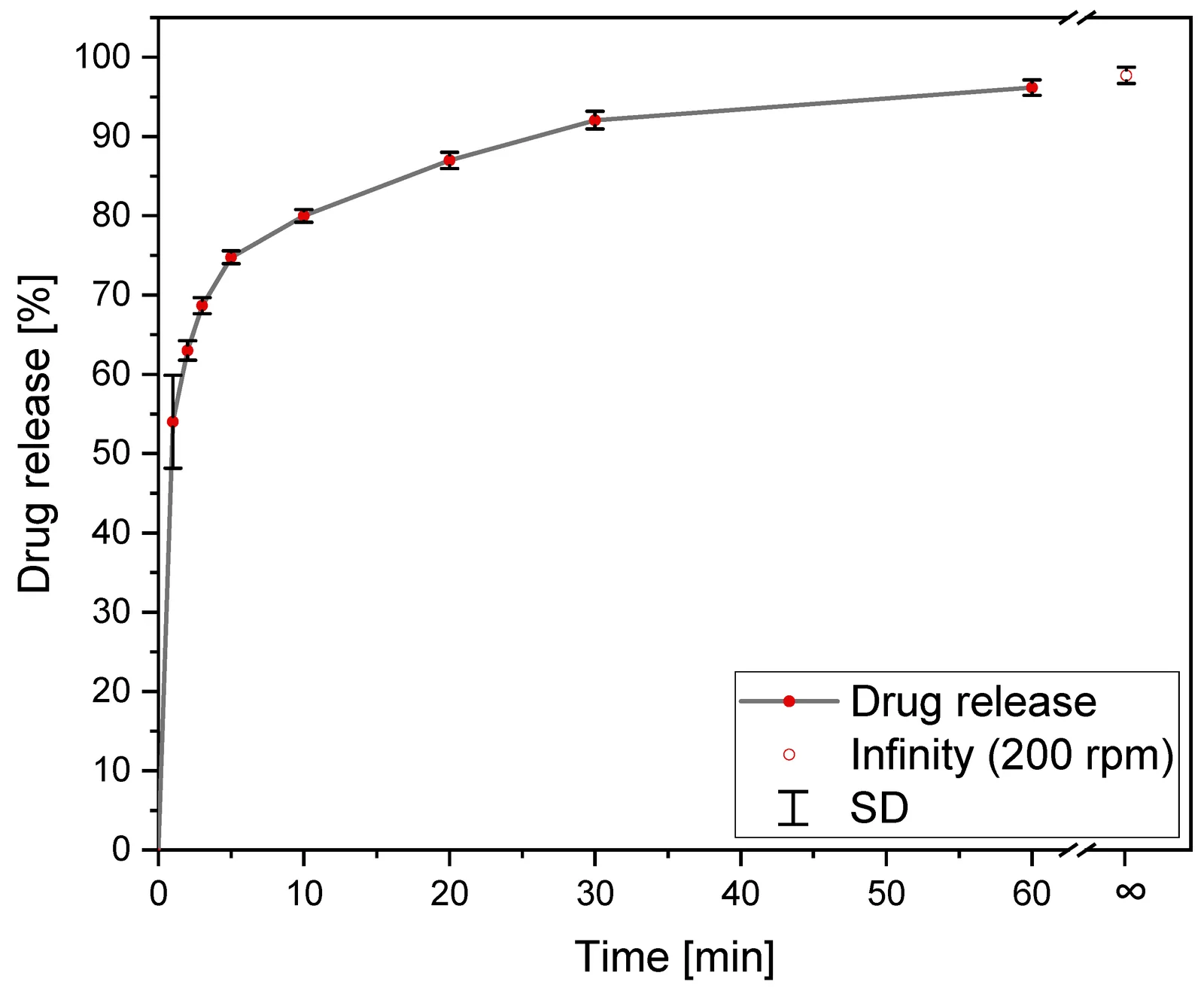
Dissolution of 1 mg CHILD-IVITAB tablets in artificial saliva fluid (pH 6.8). The dots represent the mean values; error bars indicate standard deviation (SD) (n = 6).

To assess the release behavior of ivermectin from the 1 mg CHILD-IVITAB ODT, dissolution testing was performed in an artificial saliva fluid medium (AFS) using the paddle apparatus method (n = 6). Figure 2 shows the dissolution profile of the 1 mg CHILD-IVITAB ODT, evaluated in AFS (pH 6.8) containing 0.5% SLS using the paddle apparatus method (n = 6). More than 50% of the drug was released within 1 min and approximately 80% within 10 min, consistent with the rapid disintegration of the tablet. The profile showed a rapid initial phase between 0 and 10 min, followed by a markedly slower apparent release between 10 and 60 min. This second phase is interpreted as a hydrodynamic artefact arising from cone formation at the base of the vessel, where the insoluble TIP matrix accumulates beneath the paddle due to the vessel geometry [24]. Consistent with this interpretation, increasing the paddle speed to 200 rpm for 5 min disrupted the cone and yielded a final recovery of 97.7% of the label claim. This final sample corresponds to the infinity point defined in USP <1092> and is reported as a mass-balance and recovery determination rather than as a dissolution time point [19].

### 3.2. Study Participants and Trial Design

**Figure 3:**
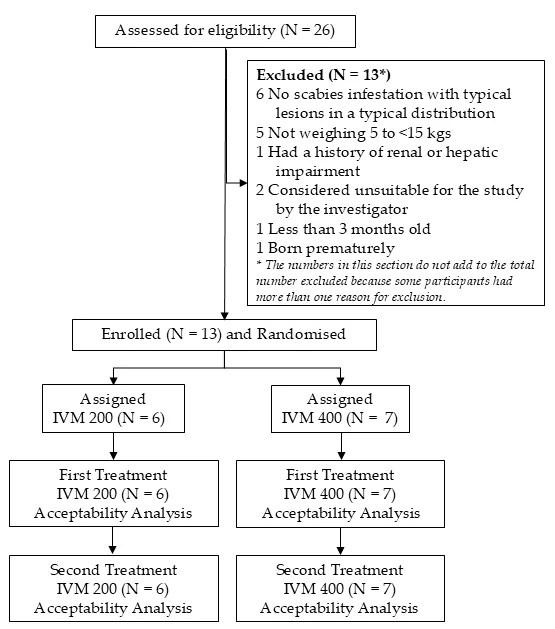
Participant flow chart.

Due to difficulties with subject recruitment, only 13 participants were enrolled from 25 February to 30 November 2025. Study recruitment ended when CHILD-IVITAB tablets expired on 30 November 2025. All subjects completed both D0 and D7 drug administrations with no withdrawals, for a total of 26 acceptability evaluations (Figure 3). Of those participants, eight (61.5%) were male and five (38.5%) were female, while six (46%) were in the 200 μg/kg group and seven (54%) were in the 400 μg/kg group. Children ranged from 4.6 to 27 months, with five (38%) younger than 1 year old and eight (62%) aged 1 year or older. There were no significant differences in sex, age, or weight between the two dose groups (Table 2). No coughing, gagging, choking, vomiting, oral irritation, or aspiration concerns were observed with CHILD-IVITAB administration. No spitting or excessive drooling, which would have led to loss of study drug, was observed.

### 3.3. Acceptability

**Figure 4:**
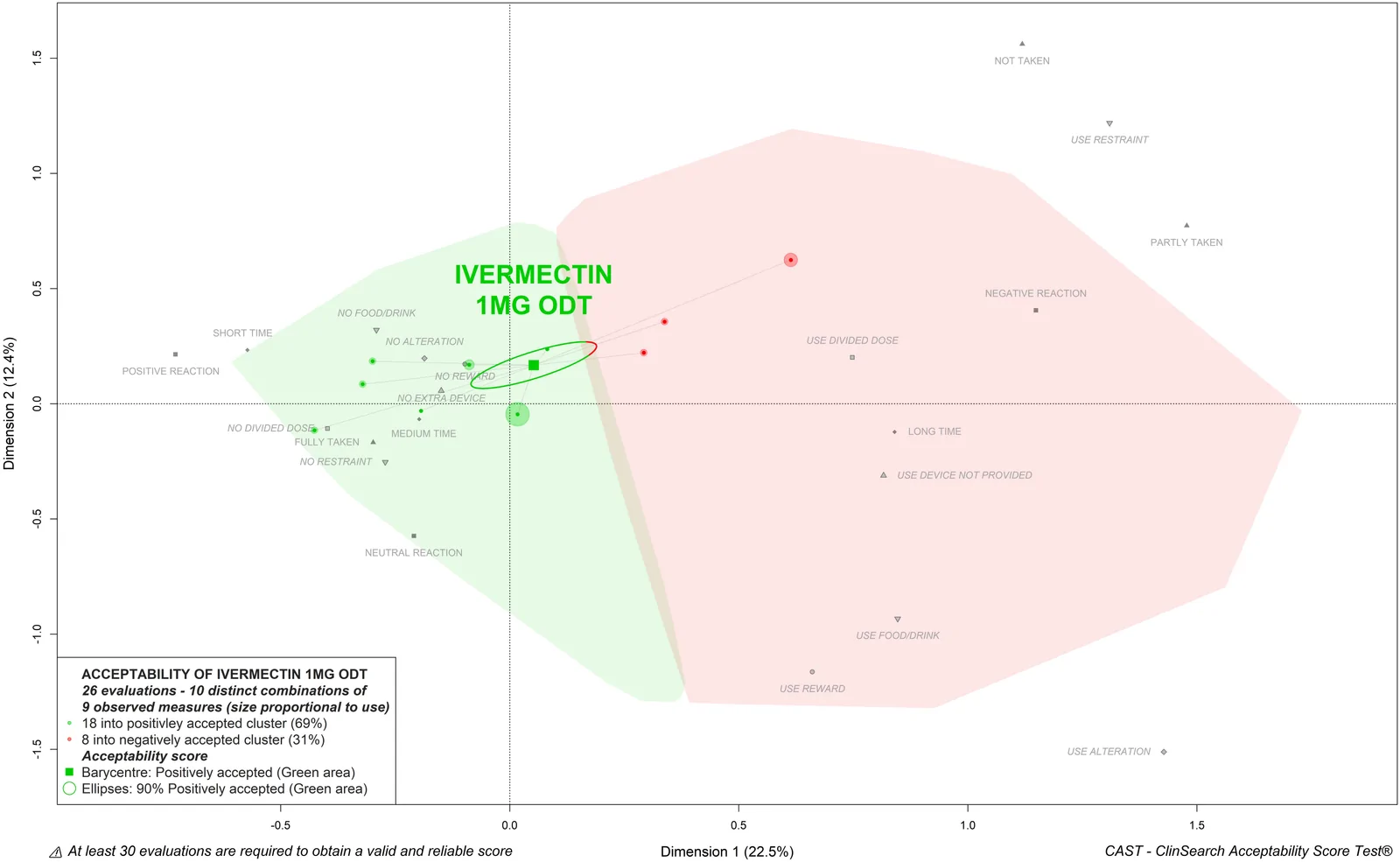
Position of CHILD-IVITAB on the CAST framework. Barycenter and 90% confidence ellipse of all evaluations (n = 26 [13 participants]).

The barycenter of all CHILD-IVITAB evaluations was located in the positive zone (green) of the acceptability reference framework (Figure 4). However, CHILD-IVITAB could not be formally classified as “positively accepted”, as approximately 10% of the 90% confidence interval ellipse extended into the negative zone (red). This is primarily attributable to the small subject sample size, producing 26 evaluations in only 13 participants, which was below the threshold of 30 evaluations required for a reliable classification.

The dose was fully taken in all 26 evaluations (100%). The majority of children showed a neutral (46%) or positive (27%) reaction during administration, while negative reactions were recorded for 27% of administrations. No evaluations required dosage form alteration, co-administration with food or drink, use of an extra administration device, or promise of a reward. Physical restraint, defined in this paper as holding the child in a specific position for administration, was used in 23% of cases. Preparation and administration time was classified as long (i.e., more than 2.5 min) in 77% of evaluations, which was related to the dose-splitting protocol requiring alternating tablet administration between opposite cheek pouches and waiting for tablet disintegration prior to subsequent administration. Indeed, nine (69%) subjects required three or more CHILD-IVITAB tablets.

Preparation time ranged from 10 s to 1 min 20 s, with a median of 20 s. Administration time ranged from 1 min 10 s to 4 min, with a median of 2 min 30 s. The combined preparation and administration time ranged from 1 min 20 s to 4 min 20 s, with a median of 3 min 10 s.

When compared against the average acceptability of conventional tablets in children of the same age from the CAST framework reference dataset, CHILD-IVITAB showed higher rates of full dose intake (100% vs. 55%), fewer negative reactions (27% vs. 58%), and complete absence of dosage form alteration (0% vs. 82%), co-administration with food or drink (0% vs. 81%), and use of extra devices (0% vs. 49%) (Table 3; Supplemental Table S1, Supplemental Figure S1). The differences remained significant when the first (D0) and second (D7) administrations were evaluated independently relative to the reference dataset.

No significant difference in acceptability scores was observed between the first (D0) and second (D7) administrations (Supplemental Figure S2). Most subjects showed no change, or only minor variations, in the constituting variables over time (Supplemental Table S2). Changes were observed only for Patient reaction and Restraint. For Patient reaction, scores remained stable in 6/13 subjects, worsened in 5/13, and improved in 2/13, with no statistically significant difference between D0 and D7 (paired Wilcoxon signed-rank test, V = 21, *p* = 0.240). Similarly, Restraint status remained unchanged in 11/13 subjects, with one subject switching from “No restraint” to “Use restraint” and one showing the opposite change; this difference was not significant (exact McNemar test, *p* = 1.000).

**Figure 5:**
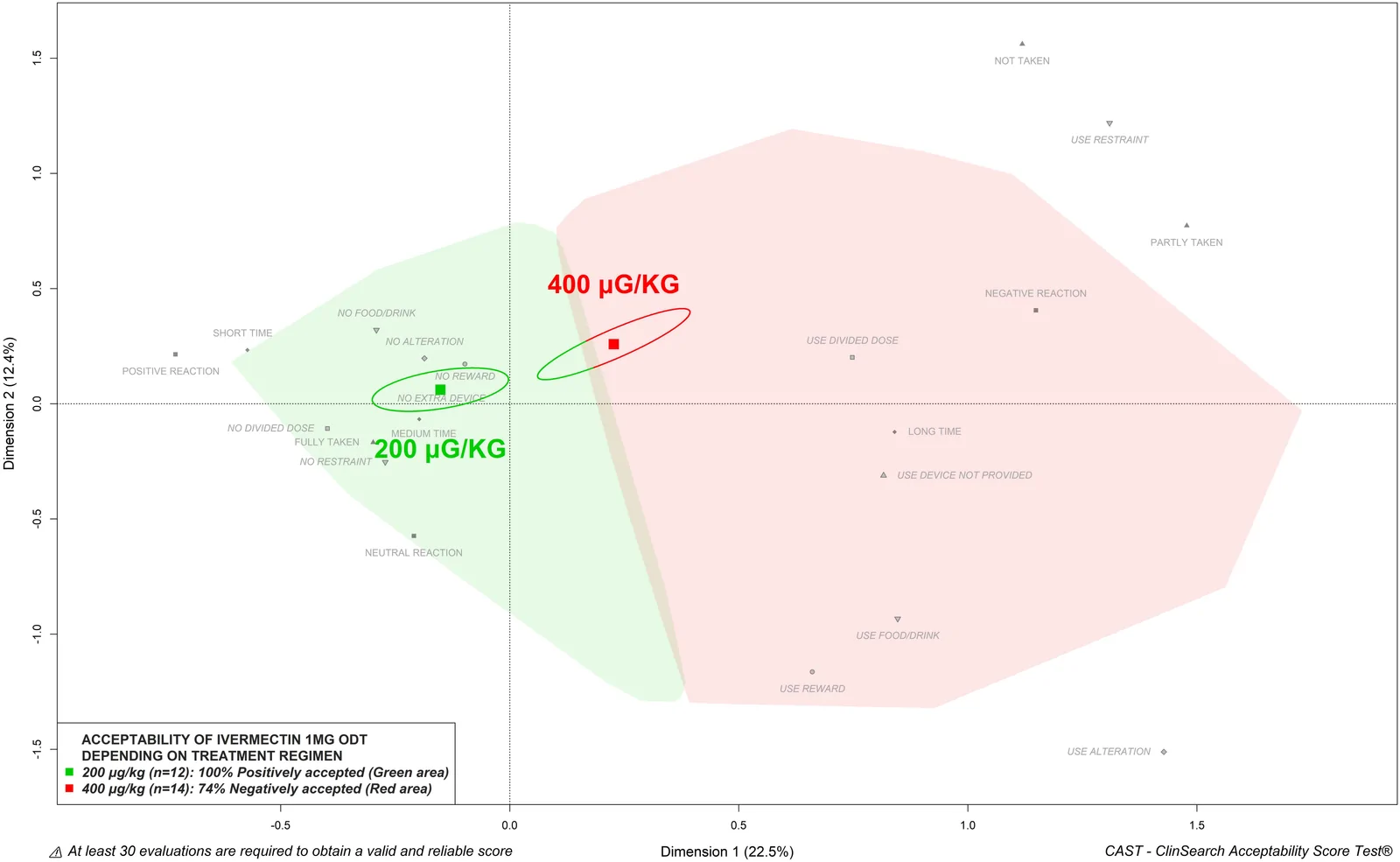
Acceptability of CHILD-IVITAB by treatment regimen on the CAST framework. Barycenter and 90% confidence ellipse are shown for each group 200 μg/kg (n = 12 [6 participants]) and 400 μg/kg (n = 14 [7 participants]).

A trend toward greater acceptability was observed for the 200 μg/kg regimen compared to 400 μg/kg (Figure 5). This could be due to shorter preparation and administration times and fewer divided doses (Supplemental Table S3), reflecting a lower number of tablets required (Table 2). Indeed, four (67%) of six subjects in the 200 μg/kg regimen received less than three CHILD-IVITAB ODTs, while all seven (100%) of subjects in the 400 μg/kg regimen received three or more CHILD-IVITAB ODTs (Table 2). It could also be related to the higher proportion of negative reactions and more frequent use of restraint in the 400 μg/kg group, although this should be interpreted cautiously due to the small sample size (Supplemental Table S3). The sample size was insufficient to meaningfully evaluate the impact of sex or age on CHILD-IVITAB acceptability as the number subgroup evaluations was not sufficient for females or children <1 year old.

## 4. Discussion

The CHILD-IVITAB (1 mg) formulation showed a favorable combination of high mechanical strength (1.87 ± 0.16 MPa) and rapid in vitro disintegration (18.3 ± 1.1 s) (Table 1), enabled by the unique properties of the TIP microcapsules. The porous shell allows for fast water uptake, while the compacted calcium phosphate delivers high structural integrity [9]. The formulation of tablets with TIP as a carrier allows for the splitting of the API into small, isolated domains of loaded TIP particles. This removes formulation property dependency on physiochemical properties of the drug substance [10]. This approach enables consistent compaction behavior and predictable water uptake. Ivermectin is classified as a Biopharmaceutics Classification System Class II (high permeability and low solubility) compound, with absorption limited by solubility rather than permeability [25].

The disintegration and dissolution methods used in this paper were developed as quality-control and discriminatory tests, and do not model the conditions in the pediatric oral cavity. It should be noted that in vitro disintegration under pharmacopeial conditions can differ from disintegration in the oral cavity, where mechanical stress from the tongue and biorelevant saliva volumes create a physiologically different environment [26]. Rapid release was observed in the dissolution testing in AFS, with >50% released within 1 min (Figure 2). Independently, a recent phase I crossover study in healthy adults who received a single 12 mg dose of CHILD-IVITAB reported a shorter median T_max_ than Stromectrol^®^ (3.0 h [range of 2.0–4.0] versus 4.0 h [range of 2.0–5.0]; *p* = 0.004).

The CAST^®^ reference framework showed that the barycenter of all CHILD-IVITAB evaluations was located in the “positive zone”. The required dose was fully taken in all 26 evaluations, which is a notable result in such a young patient group (4.6 to 27 months of age), where acceptability issues are common. In an exploratory comparison with conventional tablets in children of the same age group from the dataset that led to the reference framework, CHILD-IVITAB was associated with a higher proportion of full dose intake, fewer negative reactions, and no need for dosage form alterations or taste masking strategies. This demonstrates the advantages of the TIP technology, where the rapid disintegration times eliminate the need for swallowing of a solid tablet, and the TIP microcapsules provide enhanced taste masking, as the drug is contained within the microcapsules. Furthermore, the small tablets (5 mm diameter, 1.14 mm thickness) of only 40 mg size provide a child-appropriate size, which is well-suited for buccal placement even in young children. It should be noted that the CAST reference dataset has limitations, as the reference evaluations covered different drugs across multiple therapeutic areas. As such, the present preliminary findings do not allow conclusions to be drawn regarding the relative acceptability of CHILD-IVITAB compared with currently available ivermectin tablet formulations.

One practical challenge encountered in the clinic was the long preparation and administration time, which was observed in 77% of evaluations. This is primarily explained by the dose-splitting protocol, where tablets are placed alternately in the left and right buccal pouches. As such, a better acceptability was observed for the 200 μg/kg regimen using one to three tablets compared to 400 μg/kg using two to six tablets. This directly reflects the lower number of tablets required, leading to faster administration. Although this should be interpreted cautiously due to the small sample size, the higher dose group showed more negative reactions and more physical restraint during the administration. These findings suggest that increasing the amount of ivermectin per tablet to minimize the number of tablets per dose could improve the acceptability further. Nine out of thirteen children required three or more tablets. A CHILD-IVITAB 2 mg tablet would reduce the 400 μg/kg regimen to one to three tablets, matching the lower tablet count of the better accepted 200 μg/kg arm (Figure 4). The TIP technology readily supports this approach, as ivermectin drug loads of up to 45% (*v*/*v*) have been demonstrated previously [10], far exceeding the drug load used in this formulation, meaning that substantially higher doses are supported while maintaining the same tablet size. The 1 mg strength should therefore be understood as a dose-ranging tool appropriate to a phase IIb design across a 5 to <15 kg weight range and two dose levels, rather than as the intended deployment formulation. An oral ivermectin dose of 400 µg/kg appears to be safe in children <15 kg and ≥2 years of age [27], with preliminary evidence from the recently completed EPIC-15 and Ivermectin Safety in Small Children (NCT04332068) trials indicating this dose is safe down to ≥2 months of age.

The main limitation of this study is the small sample size of 13 children, compared to the planned number of 66. This was caused by difficulties in recruiting eligible subjects within the shelf life of the clinical batch, which was only 12 months. Due to the limited sample size (n < 30), the resulting CAST score cannot be considered sufficiently robust and should be interpreted as a trend only. CHILD-IVITAB cannot be considered as “positively accepted”, as the 90% confidence ellipses partially extended into the negative zone. All statistical analyses were considered exploratory due to the limited sample size. No adjustment for multiplicity was applied. With a larger number of participants, we would expect to achieve improved results and a narrower ellipse around the barycenter, which itself was located in the positive zone. Furthermore, the sample size was too small to reliably assess the influence of sex or age on acceptability, and the statistical power for other subgroup comparisons was limited. Despite these constraints, the key findings of 100% dose completion, absence of dosage form alterations, and better performance than conventional tablets provide promising preliminary evidence for this novel CHILD-IVITAB formulation use in children <15 kg.

Another limitation is that long-term stability of the clinical formulation under tropical storage conditions was not established experimentally. The assigned 12-month shelf life was based on the literature [11], rather than dedicated stability testing. Formal long-term and accelerated stability studies, including conditions relevant to ICH climatic Zone IVb, are needed to support large-scale deployment in MDA settings.

## 5. Conclusions

The CHILD-IVITAB tablets demonstrated a favorable combination of mechanical strength and rapid in vitro disintegration, fast drug release, and excellent uniformity of mass and dosage units. These results support the potential of CHILD-IVITAB as a child-friendly oral ivermectin formulation for a vulnerable population group that is currently excluded from ivermectin treatment and MDA programs. Larger studies are needed to confirm these preliminary findings and to formally classify CHILD-IVITAB as “positively accepted”. This would enable the inclusion of children <15 kg in ivermectin MDA programs for scabies and other neglected tropical diseases, addressing a critical gap in global pediatric health.

## Figures and Tables

**Table 1:** Summary of pharmaceutical characterization of the 1 mg CHILD-IVITAB ODT.

Parameter (Unit)	n	Mean ± SD	Range	RSD (%)	Acceptance Criterion	Compliance
Tensile strength (MPa)	6	1.87 ± 0.16	1.77–2.12	8.6	>1.7 MPa	Pass
Tablet thickness (mm)	6	1.14	1.13–1.16	0.7%	<1.5 mm	Pass
Disintegration time (s)	6	18.3 ± 1.1	16.5–19.6	6.1	≤180 s (Ph. Eur. 0478)	Pass
Uniformity of mass (mg)	950	40.55 ± 0.46	38–42	1.1	±10% (Ph. Eur. 2.9.5)	Pass
Content uniformity (mg)	10	1.00 ± 0.02	0.98–1.05	2.0	AV ≤ 15.0 (Ph. Eur. 2.9.40)	Pass (AV = 5.5)

**Table 2:** Comparison of ivermectin 200 and 400 µg/kg groups.

	200 µg/kg (6 Participants)	400 µg/kg (7 Participants)	Statistical Test ^a^ (*p*-Value)
**Sex**			F: *p* = 1
Male	4 (66.7)	4 (57.1)	
Female	2 (33.3)	3 (42.9)	
**Age group**			F: *p* = 0.59
<1 y	3 (50)	2 (28.6)	
≥1 y	3 (50)	5 (71.4)	
**Weight group**			F: *p* = 0.59
5–9.9 kg	2 (33.3)	4 (57.1)	
10–14.9 kg	4 (66.7)	3 (42.9)	
**Number of prescribed ODT**			F: *p* = 0.21
1	2 (33.3)	0 (0)	
2	2 (33.3)	0 (0)	
3	2 (33.3)	3 (42.9)	
4	0 (0)	2 (28.6)	
5	0 (0)	1 (14.3)	
6	0 (0)	1 (14.3)	

^a^ F: Fisher’s Exact Test *p*-value.

**Table 3:** Ivermectin orodispersible tablet (ODT) compared with conventional tablets regardless of treatment: observer-reported outcomes.

	Ivermectin ODT (n = 26 [13 Participants])	Tablets (n = 80)
**Result intake**		
Fully taken	26 (100)	44 (55)
Partly taken	0 (0)	36 (45)
Not taken	0 (0)	0 (0)
**Patient reaction**		
Positive	7 (27)	21 (26)
Neutral	12 (46)	13 (16)
Negative	7 (27)	46 (58)
**Preparation and administration time**		
Short	0 (0)	3 (4)
Medium	6 (23)	23 (29)
Long	20 (77)	54 (68)
**Divided dose**		
No divided dose	4 (15)	26 (32)
Use divided dose	22 (85)	54 (68)
**Alteration**		
No alteration	26 (100)	14 (18)
Use alteration	0 (0)	66 (82)
**Food/drink**		
No food/drink	26 (100)	15 (19)
Use food/drink	0 (0)	65 (81)
**Extra device**		
No extra device	26 (100)	41 (51)
Use device not provided	0 (0)	39 (49)
**Reward**		
No reward	26 (100)	69 (86)
Use reward	0 (0)	11 (14)
**Restraint**		
No restraint	20 (77)	47 (59)
Use restraint	6 (23)	33 (41)

## Data Availability

Data presented in this study is contained within the article and Supplementary Material. Further inquiries for data, trial protocol, and statistical analyses can be directed to the corresponding author.

## Supplementary Materials

The following supporting information can be downloaded at: https://www.mdpi.com/article/10.3390/pharmaceutics18091147/s1, Figure S1: Ivermectin ODT for scabies compared with conventional tablets regardless of treatment: acceptability scores; Figure S2: The influence of evaluation timepoint on acceptability of ivermectin ODT: acceptability scores; Table S1: Ivermectin ODT for scabies compared with conventional tablets regardless of treatment: Subject characteristics; Table S2: The influence of evaluation timepoint on acceptability of ivermectin ODT: observer-reported outcomes; Table S3: The influence of treatment regimen on acceptability of ivermectin ODT: Observer-reported outcomes.
