# Supplementary material for: CHILD-IVITAB, a Novel Orodispersible Ivermectin Formulation—Pharmaceutical Evaluation and Clinical Acceptability in Children <15 kg
Source: Pharmaceutics. 2026 Sep 11;18(9):1147. doi: 10.3390/pharmaceutics18091147 (PMC13610006; doi:10.3390/pharmaceutics18091147)
Supplement: Supplementary file 1 [file pharmaceutics-18-01147-s001.zip › pharmaceutics-4438029-supplementary.pdf]

Supplementary Materials

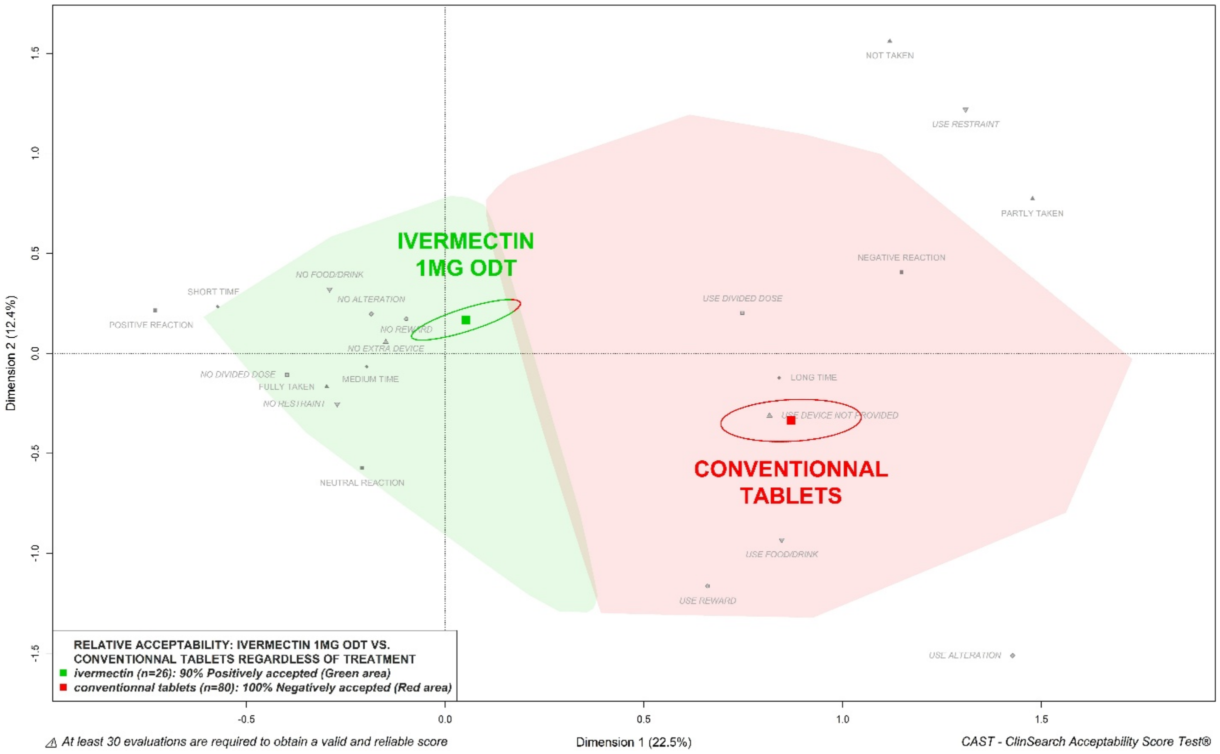

Supplemental Figure S1. Ivermectin ODT for scabies compared with conventional tablets regardless of treatment: acceptability scores.

Supplemental Table S1. Ivermectin ODT for scabies compared with conventional tablets regardless of treatment: Subject characteristics.

	**Ivermectin ODT** (13 participants)	**Tablets** (80 patients)	**Statistical test[a] (p-value)**
**Sex**			χ2: P=.96
Male	8 (62)	45 (56)	
Female	5 (38)	35 (44)	
**Age group**			χ2: P=1
<1y	5 (38)	31 (39)	
≥1y	8 (62)	49 (61)	
**Country**			χ2: P<.001 *
Brazil	13 (100)	0 (0)	
India	0 (0)	35 (44)	
Morocco	0 (0)	37 (46)	
**ATC 1st level (main anatomical or pharmacological groups)[b]**			χ2: P<.001 *
Alimentary tract and metabolism	0 (0)	13 (16)	
Anti-infectives for systemic use	0 (0)	14 (18)	
Antiparasitic products, insecticides, and repellents	13 (100)	1 (1)	
Nervous system	0 (0)	28 (35)	

[a] *χ2: Pearson's Chi-squared Test p-value; *: significant (p<0.05);* [b] *Categories with n≥10*

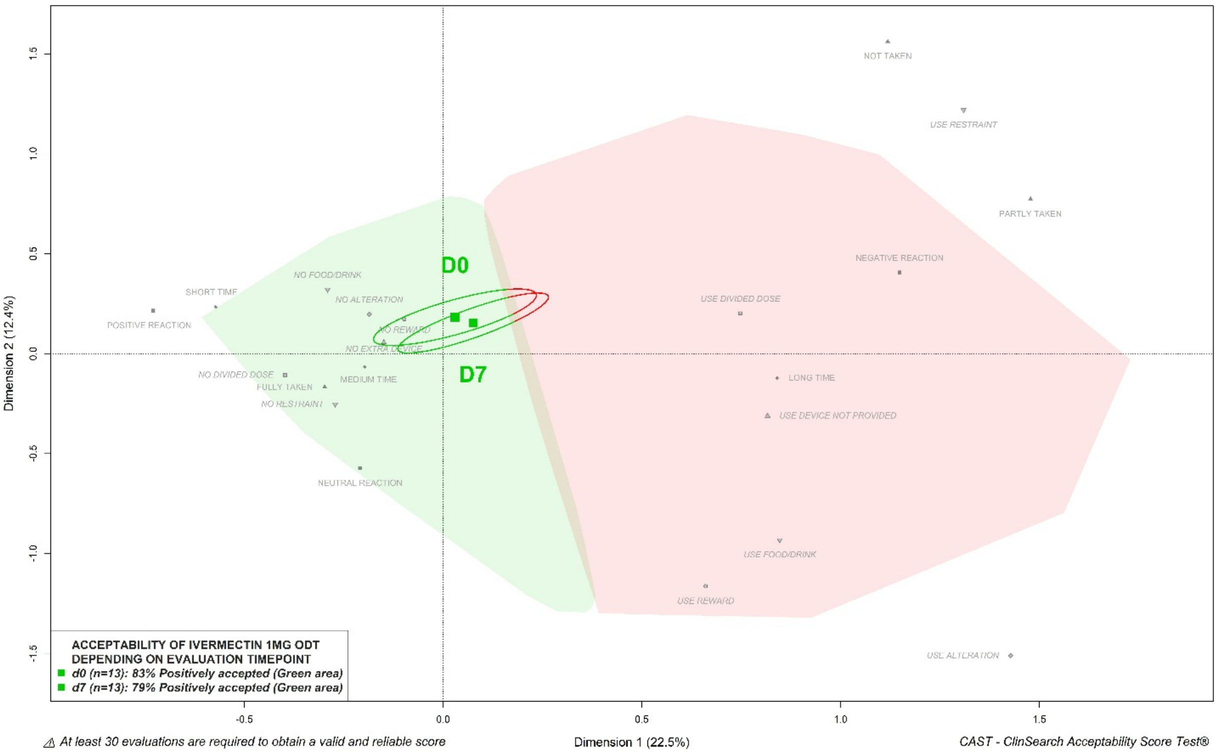

Supplemental Figure S2. The influence of evaluation timepoint on acceptability of ivermectin ODT: acceptability scores.

Supplemental Table S2. The influence of evaluation timepoint on acceptability of ivermectin ODT: observer-reported outcomes.

	**D0**<br>(n = 13)	**D7**<br>(n = 13)
**Result intake**		
Fully taken	13 (100)	13 (100)
Partly taken	0 (0)	0 (0)
Not taken	0 (0)	0 (0)
**Patient reaction**		
Positive	5 (38)	2 (15)
Neutral	5 (38)	7 (54)
Negative	3 (23)	4 (31)
**Preparation and administration time**		
Short	0 (0)	0 (0)
Medium	3 (23)	3 (23)
Long	10 (77)	10 (77)
**Divided dose**		
No divided dose	2 (15)	2 (15)
Use divided dose	11 (85)	11 (85)
**Alteration**		
No alteration	13 (100)	13 (100)
Use alteration	0 (0)	0 (0)
**Food/drink**		
No food/drink	13 (100)	13 (100)
Use food/drink	0 (0)	0 (0)

**Extra device**		
No extra device	13 (100)	13 (100)
Use device not provided	0 (0)	0 (0)
**Reward**		
No reward	13 (100)	13 (100)
Use reward	0 (0)	0 (0)
**Restraint**		
No restraint	10 (77)	10 (77)
Use restraint	3 (23)	3 (23)

Supplemental Table S3. The influence of treatment regimen on acceptability of ivermectin ODT: Observer-reported outcomes.

	200 µg/kg		400 µg/kg	
	**D0** (n = 6)	**D7** (n = 6)	**D0** (n = 7)	**D7** (n = 7)
**Result intake**				
Fully taken	6 (100)	6 (100)	7 (100)	7 (100)
Partly taken	0 (0)	0 (0)	0 (0)	0 (0)
Not taken	0 (0)	0 (0)	0 (0)	0 (0)
**Patient reaction**				
Positive	3 (50)	1 (17)	2 (29)	1 (14)
Neutral	3 (50)	4 (67)	2 (29)	3 (43)
Negative	0 (0)	1 (17)	3 (43)	3 (43)
**Preparation and administration time**				
Short	0 (0)	0 (0)	0 (0)	0 (0)
Medium	3 (50)	3 (50)	0 (0)	0 (0)
Long	3 (50)	3 (50)	7 (100)	7 (100)
**Divided dose**				
No divided dose	2 (33)	2 (33)	0 (0)	0 (0)
Use divided dose	4 (67)	4 (67)	7 (100)	7 (100)
**Alteration**				
No alteration	6 (100)	6 (100)	7 (100)	7 (100)
Use alteration	0 (0)	0 (0)	0 (0)	0 (0)
**Food/drink**				
No food/drink	6 (100)	6 (100)	7 (100)	7 (100)
Use food/drink	0 (0)	0 (0)	0 (0)	0 (0)
**Extra device**				
No extra device	6 (100)	6 (100)	7 (100)	7 (100)
Use device not provided	0 (0)	0 (0)	0 (0)	0 (0)
**Reward**				
No reward	6 (100)	6 (100)	7 (100)	7 (100)
Use reward	0 (0)	0 (0)	0 (0)	0 (0)
**Restraint**				
No restraint	6 (100)	5 (83)	4 (57)	5 (71)
Use restraint	0 (0)	1 (17)	3 (43)	2 (29)
